# Supplementary material for: Empirical methods for controlling false positives and estimating confidence in ChIP-Seq peaks
Source: BMC Bioinformatics. 2008 Dec 5;9:523. doi: 10.1186/1471-2105-9-523 (PMC2628906; doi:10.1186/1471-2105-9-523)
Supplement: Additional File 1 — A variety of html documents from the USeq web site detailing the available applications, their best usage, output file type descriptions, command line menus, etc. [file 1471-2105-9-523-S1.zip › USeqUserGuides/JavaDoc.zip!/JavaDoc/util/bio/wrappers/SimpleBlast.html]

SimpleBlast


|  |  |  |
| --- | --- | --- |
| **Overview** \| **Package** \| **Class** \| **Use** \| **Tree** \| **Deprecated** \| **Index** \| **Help** | |  |
| **PREV CLASS**   NEXT CLASS | **FRAMES**    **NO FRAMES**     **All Classes** |  |
| SUMMARY: NESTED \| FIELD \| CONSTR \| METHOD | DETAIL: FIELD \| CONSTR \| METHOD |  |


---


## util.bio.wrappers Class SimpleBlast

```
java.lang.Object
  util.bio.wrappers.SimpleBlast
```

---

``` public class SimpleBlast extends java.lang.Object ```

Wrapper for bl2seq blast.

---

**Constructor Summary**

`SimpleBlast(java.lang.String bl2seq)`


| **Method Summary** | |
| --- | --- |
| `java.lang.String[]` | `blastIt(java.lang.String seq1File, java.lang.String seq2File)` |
| `static java.util.ArrayList` | `parseBlast(java.lang.String[] data)` |
| `static BlastResult[]` | `parseBlastGeneral(java.lang.String[] data)`             Parses blast output from blastall for dna into BlastResult objects. |
| `static java.util.ArrayList` | `parseLine(java.lang.String line, java.util.regex.Pattern pat)` |

**Methods inherited from class java.lang.Object**

`equals, getClass, hashCode, notify, notifyAll, toString, wait, wait, wait`

**Constructor Detail**

### SimpleBlast

```
public SimpleBlast(java.lang.String bl2seq)
```


**Method Detail**

### blastIt

```
public java.lang.String[] blastIt(java.lang.String seq1File,
                                  java.lang.String seq2File)
```

---


### parseBlast

```
public static java.util.ArrayList parseBlast(java.lang.String[] data)
```

---


### parseLine

```
public static java.util.ArrayList parseLine(java.lang.String line,
                                            java.util.regex.Pattern pat)
```

---


### parseBlastGeneral

```
public static BlastResult[] parseBlastGeneral(java.lang.String[] data)
```

:   Parses blast output from blastall for dna into BlastResult objects.


---


|  |  |  |
| --- | --- | --- |
| **Overview** \| **Package** \| **Class** \| **Use** \| **Tree** \| **Deprecated** \| **Index** \| **Help** | |  |
| **PREV CLASS**   NEXT CLASS | **FRAMES**    **NO FRAMES**     **All Classes** |  |
| SUMMARY: NESTED \| FIELD \| CONSTR \| METHOD | DETAIL: FIELD \| CONSTR \| METHOD |  |


---
